# Effects of Resistant-Starch-Encapsulated Probiotic Cocktail on Intestines Damaged by 5-Fluorouracil

**DOI:** 10.3390/biomedicines12081912

**Published:** 2024-08-20

**Authors:** Jui-Ling Wang, Chin-Hsing Yeh, Shih-Hung Huang, Lawrence Shih-Hsin Wu, Miles Chih-Ming Chen

**Affiliations:** 1 Animal Testing Division, National Applied Research Laboratories, National Laboratory Animal Center, Tainan 744, Taiwan; jlw1975@narlabs.org.tw; 2 Fecula Biotech Co., Ltd., Tainan 744, Taiwan; sandy@fecula.com.tw (C.-H.Y.); xhshkimo@yahoo.com.tw (S.-H.H.); 3 Graduate Institute of Biomedical Sciences, China Medical University, Taichung 404, Taiwan

**Keywords:** probiotics, 5-fluorouracil, resistant starch, microbiota dysbiosis

## Abstract

Probiotics and prebiotics have gained attention for their potential health benefits. However, their efficacy hinges on probiotic survival through the harsh gastrointestinal environment. Microencapsulation techniques provide a solution, with resistant starch (RS)-based techniques showing promise in maintaining probiotic viability. Specifically, RS-encapsulated probiotics significantly improved probiotic survival in gastric acid, bile salts, and simulated intestinal conditions. This study investigated the effects of a resistant-starch-encapsulated probiotic cocktail (RS-Pro) in the context of 5-fluorouracil (5-FU) chemotherapy, which frequently induces microbiota dysbiosis and intestinal mucositis. Female BALB/c mice were divided into three groups: a 5-FU group, a 5-FU+Pro group receiving free probiotics, and a 5-FU+RS-Pro group receiving RS-encapsulated probiotics. After 28 days of treatment, analyses were conducted on fecal microbiota, intestinal histology, peripheral blood cell counts, and body and organ weights. It was revealed by 16S rRNA MiSeq sequencing that 5-FU treatment disrupted gut microbiota composition, reduced microbial diversity, and caused dysbiosis. RS-Pro treatment restored microbial diversity and increased the population of beneficial bacteria, such as *Muribaculaceae,* which play roles in carbohydrate and polyphenol metabolism. Furthermore, 5-FU administration induced moderate intestinal mucositis, characterized by reduced cellularity and shortened villi. However, RS-Pro treatment attenuated 5-FU-induced intestinal damage, preserving villus length. Mild leukopenia observed in the 5-FU-treated mice was partially alleviated in 5-FU+Pro and 5-FU+RS-Pro groups. These findings suggest that RS-Pro may serve as an adjunct to chemotherapy, potentially reducing adverse effects and improving therapeutic outcomes in future clinical applications.

## 1. Introduction

Human gut microbiota plays a pivotal role in shaping various aspects of human physiology, including metabolism and immune functions. They help to promote gut integrity, protect against pathogens, and maintain the overall equilibrium of the host defense system. Dysbiosis of the gut microbiome has been linked to chronic inflammation and the development of conditions such as colorectal cancer, obesity, inflammatory bowel disease (IBD), allergies, and autoimmune disorders [1,2,3]. For instance, patients with IBD often exhibit an increased abundance of *Bacteroides* and Enterobacteriaceae populations alongside a decrease in *Lactobacillus* and *Bifidobacterium* populations [4].

The chemotherapy drug 5-fluorouracil (5-FU) has been the cornerstone of colorectal cancer treatment since 1962 [5,6]. However, its use is associated with various adverse effects on the gastrointestinal, cardiovascular, and hematopoietic systems [7,8,9,10]. Studies have highlighted the effects of 5-FU on gut microbiota, including its role in disrupting the delicate balance among resident microorganisms and causing intestinal inflammation [11,12,13].

Probiotics are living microorganisms that, when ingested in adequate quantities, confer health benefits to the host by modulating gut microbiota [1,14,15]. Research has helped to clarify the mechanisms underlying how probiotics affect intestinal health and prevent diseases. These mechanisms include the inhibition of pathogenic activities through the production of antibacterial substances, the competitive inhibition of pathogen and toxin adherence to the intestinal epithelium, the regulation of immune responses by dampening proinflammatory responses and enhancing anti-inflammatory immunity, and the stabilization of the gut microbial environment and intestinal permeability to counteract inflammation [16,17,18,19]. Nevertheless, the viability of orally administered probiotics is challenged by the harsh conditions in the stomach associated with the presence of gastric acid, bile salts, and digestive enzymes, limiting the likelihood of them reaching their functional site in the gastrointestinal tract [20,21].

Resistant starch (RS) is a type of starch that can resist digestion by amylases in the small intestine and pass to the colon, where it undergoes fermentation by gut microbiota. Because of its ability to reach the colon, RS is a promising candidate for enteric delivery, allowing for the release of encapsulated bacterial cells in the colon. Additionally, RS exhibits prebiotic functionality, serving as a substrate for probiotic and gut bacteria in the large intestine [22,23,24,25,26,27]. When undigested RS reaches the colon, it undergoes fermentation, producing short-chain fatty acids such as acetate, propionate, and butyrate, which are known for their anti-inflammatory properties [28]. Venkataraman et al. (2016) demonstrated the positive effects of RS on the microbiome and in particular the association of RS with increased butyrate production in the large intestine. Several studies have highlighted the crucial roles of butyrate in human gut health, including its effects on reducing inflammation, reducing colon cancer risk, and improving gut barrier function [29,30]. Given that RS is regarded as healthy for humans and animals and is also a prebiotic, encapsulation is the optimal method for achieving prebiotic–probiotic symbiosis. Sultana et al. (2000) reported that the addition of commercial RS improved the encapsulation of viable bacteria (*Lactobacillus acidophilus* and *Bifidobacterium* spp.) in yogurt [31,32].

In the present study, we employed several widely used probiotics as a probiotic cocktail (denoted as Pro), namely, *Bifidobacterium longum*, *Lactobacillus casei*, *Lactobacillus rhamnosus*, *Streptococcus thermophilus*, and *Clostridium butyricum* (a butyrate-producing human gut symbiont). In addition, we used RS as an encapsulation medium for a probiotic cocktail (denoted as RS-Pro) and subsequently explored the protective potential and underlying mechanisms of Pro and/or RS-Pro to against 5-FU-induced adverse effects in a pilot study.

## 2. Materials and Methods

### 2.1. Probiotic Cocktail and RS-Based Encapsulation Technique

**Figure 1:**
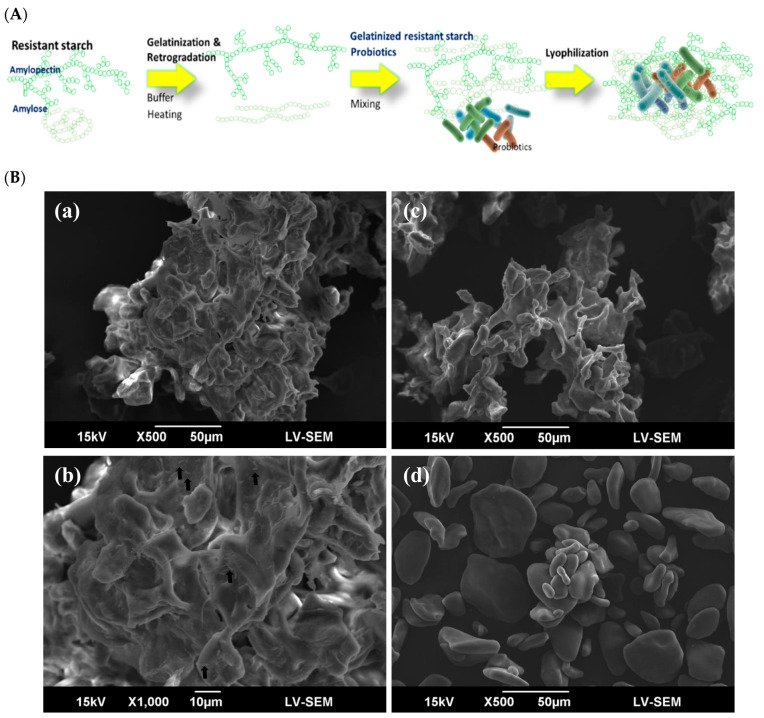
The manufactural process and SEM images of resistant-starch-encapsulated probiotic cocktails (RS-Pro). (**A**) Schematic diagram of the resistant-starch-based encapsulation technique (RS-BET) process to produce resistant-starch-encapsulated probiotic cocktails (RS-Pro). (**B**) Scanning electron micrographs (SEM) of RS-Pro (**a**,**b**), RS processed by RS-BET, (**c**) and naïve RS (**d**) are shown. Black arrows indicate probiotic cells captured within the matrix.

The *B. longum*, *L. casei*, *L. rhamnosus*, and *S. thermophilus* were obtained from Glac Biotech, Tainan City, Taiwan, and *C. butyricum* was obtained from New Bellus Enterprises, Tainan City, Taiwan. A formulation of the probiotic cocktail was encapsulated using RS extracted from green bananas. In the initial stages of the encapsulation process, 20% of the RS was heated to 75 °C, thoroughly mixed, and subsequently cooled to room temperature. The probiotic cocktail was then combined with the gelatinized RS to create a paste. Subsequently, an amount of RS powder equivalent to three times the weight of the paste was added. Finally, the paste underwent lyophilization to yield the finished product (i.e., RS-Pro; Figure 1A).

### 2.2. Scanning Electron Microscope Analysis

To examine the morphology of the RS-Pro, field emission scanning electron microscopy (FESEM) was employed to characterize its surface features. Prior to scanning, dried RS-Pro samples were mounted on stubs and coated with a thin layer of gold film to enhance conductivity. The prepared samples then underwent FESEM analysis, which was conducted using a JEOL JSM-7600F microscope (JEOL Ltd., Tokyo, Japan) operating at 15 kV. During the analysis, images were recorded at both 500× and 1000× magnifications.

### 2.3. Test of Tolerance of Pro and RS-Pro to Acid and Bile Salt Solutions

A survival assessment of Pro and RS-Pro under acidic, bile salt, and simulated intestinal conditions was conducted per a modified version of the protocol developed by Jin et al. (1998) [33]. For the acid tolerance test, cultures were grown overnight in de Man, Rogosa, and Sharpe (MRS) broth (HIMEDIA) at 37 °C. Subsequently, the cultures underwent centrifugation for 10 min at 4000× *g* and 4 °C. Pellets (10% [vol/vol]) were resuspended in 0.2 M KCl–HCl buffer (pH 2.0) to conduct the control test (involving the probiotic cocktail without RS encapsulation, hereby denoted as Pro), and lyophilized RS-Pro (10% [vol/vol]) was resuspended in 0.2-M KCl–HCl buffer (pH 2.0) in parallel. Both sets of mixtures were then incubated anaerobically at 37 °C for 3 h. After incubation, 100 μL aliquots from the control test were serially diluted 10-fold in anaerobic diluent (MRS broth with 0.5 g of l-cysteine HCl per liter, pH 7.0) and plated in triplicate onto MRS agar (HIMEDIA). The resulting plates were subsequently incubated at 37 °C for 48 h under anaerobic conditions for colony enumeration.

The same procedures were performed for the bile salt tolerance test. The Pro and RS-Pro were resuspended in 0.2 M KCl–HCl buffer (pH 7.0) containing 0.3% bile salts (Sigma) and incubated at 37 °C for 3 h. Similar to the acid tolerance test, 100 μL aliquots from the samples used in the control test were serially diluted 10-fold in anaerobic diluent and plated in triplicate onto MRS agar. The resulting plates were then incubated at 37 °C for 48 h under anaerobic conditions.

To assess tolerance under simulated intestinal conditions, the Pro and RS-Pro were initially resuspended in 0.2 M KCl–HCl buffer (pH 2.0) at 37 °C for 3 h. The cultures were then subjected to centrifugation for 10 min at 4000× *g* and 4 °C. The supernatant was discarded, and the pellets were resuspended in 0.2 M KCl–HCl buffer (pH 7.0) containing 0.3% bile salts. Subsequently, they were incubated at 37 °C for an additional 3 h. One-hundred-microliter aliquots of the samples from the control test were serially diluted 10-fold in anaerobic diluent, plated in triplicate onto MRS agar, and incubated at 37 °C for 48 h under anaerobic conditions. All experiments were performed in triplicate, and colony counts were expressed as log10 colony-forming units (CFU)/mL.

### 2.4. Animal Study

Female 7-week-old BALB/cByJNarl mice weighing between 19 and 23 g were procured from the National Laboratory Animal Center (NLAC), National Applied Research Laboratories (NARLabs), Taiwan, for use in the present study. The mice were housed in a pathogen-free animal facility with controlled environmental conditions, specifically a temperature range of between 21 °C and 23 °C, a relative humidity level of between 45% and 65%, and a 12 h light/dark cycle. Food and water were provided to the mice ad libitum. The Institutional Animal Care and Use Committee of the NLAC, NARLabs, Taiwan, granted approval for this study (NLAC(TN)-109-D-003), and all procedures were conducted in the NLAC.

After 7 days of acclimatization, the mice were randomly assigned to three groups, 5-FU group, 5-FU+Pro group, and 5-FU+RS-Pro group, with each group comprising six mice. Fecal and blood samples were collected freshly from animals before receiving treatments. All mice were intraperitoneally injected with 5-FU (# 343922, Sigma-Aldrich, St. Louis, MO, USA) at a dose of 20 mg/kg, twice weekly, for a duration of 4 weeks. Mice in the 5-FU+Pro and 5-FU+RS-Pro groups additionally received Pro or RS-Pro suspension (4 × 10^8^ CFU/mouse), respectively, by oral gavage 5 days a week for 4 weeks. Mice received 300 μL of drinking water as vehicle control. During the experiment, the body weight of animals was measured twice weekly for 4 weeks. At the end of the experiment, fecal and blood samples were collected again. Fecal samples were stored at −80 °C for gut microbiota analysis and blood samples were used for blood cell counting. After the mice were euthanized, the spleen, liver, and kidney were harvested and weighed. The small intestines were collected and stored in 10% neutralized formalin solution for histological examination.

### 2.5. Gut Microbiota Analysis

The total genomic DNA of gut microbiota was meticulously extracted from fecal samples using the QIAamp Fast DNA Stool Mini Kit (QIAGEN, Valencia, CA, USA) per the manufacturer’s instructions. To assess DNA concentration and integrity, DNA samples were subjected to polymerase chain reaction (PCR) with 16S rRNA V3-V4 primers (341F/805R), followed by electrophoresis on 2% agarose gels.

The composition of gut microbiota in mice was characterized using Illumina MiSeq sequencing. The V3–V4 region of the bacterial 16S rRNA gene was amplified in each sample by using the primers 341F (5′-CCTACGGGNGGCWGCAG-3′) and 805R (5′-GACTACHVGGGTATCTAATCC-3′); each sample was assigned a unique eight-base barcode sequence for sample identification. The resulting PCR products were purified using a QIAquick PCR Purification Kit and then cleaned with AMPure XP beads (Beckman Coulter, MA, USA) per the manufacturer’s instructions. After quantification and normalization, the obtained DNA products underwent paired-end sequencing (2 × 301 bp), which was performed using the Illumina MiSeq platform in accordance with standard protocols.

Stringent criteria were applied to process the data, ultimately generating effective reads through the use of QIIME2 (https://docs.qiime2.org/2020.8/tutorials/overview/, accessed on 1 August 2024). The processing workflow followed these steps: First, paired-end sequences were scanned for adapters, which were subsequently removed. The reads were then denoised and merged into amplicon sequence variants (ASVs) without chimeras using Dada2 in QIIME2. Following this, all ASVs were classified using the Silva database (release 138). Reads classified as “Chloroplast”, “Mitochondria”, “Eukaryota”, or “unknown” (those that could not be classified at the kingdom level) were removed from further analysis.

### 2.6. Counting of Blood Cells (CBC)

The complete blood cell counting was conducted using a hematology analyzer (IDEXX, ProCyte Dx, IDEXX Laboratories, Westbrook, MA, USA).

### 2.7. Histology Analysis of Intestinal Tissue

Mouse intestinal tissue was carefully processed and fixed in 10% neutral buffered formalin, after which it was dehydrated using a sequence of increasing ethanol concentrations, cleared with xylol, and embedded in paraffin wax. Sections with a thickness of 3–4 μm were meticulously mounted on glass slides and stained by performing standard hematoxylin and eosin (HE) staining procedures. Intestinal tissue images were acquired using a TissueGnostics TissueFAXS SL (TissueGnostics GmbH, Vienna, Austria) system, and image analysis was conducted using OlyVIA version 4.1 (Olympus, Tokyo, Japan).

Morphological assessments were conducted by measuring villus length and crypt depth. For each tissue section, a rigorous selection process was employed to identify well-oriented complete villi and crypts. The average villus length and crypt depth were determined by evaluating 12 villi (from the top of the villus to the villus–crypt junction) and 12 crypts (defined as the invagination depth between adjacent villi) per the protocol described by Huang et al. (2019) [34].

### 2.8. Statistical Analysis

All parametric data were presented as the mean ± standard deviation (SD). The statistical differences between or within groups treated with two or one treatment factor(s) were analyzed using two-way or one-way analysis of variance (ANOVA), respectively. The statistical difference between groups treated with nested factor was analyzed using nested one-way ANOVA. Tukey’s multiple comparisons test was used as post hoc test. A paired *t*-test was used to compare the blood cell counts before and after treatment. Statistical analyses were conducted with Graph Pad Prism version 8.4.0 (GraphPad Software, Inc., San Diego, CA, USA).

## 3. Results

### 3.1. RS Encapsulation Enhanced Tolerance of Probiotics to Gastric Acid and Bile Salts

The primary goal of encapsulation was to shield the probiotics from adverse environmental effects. RS extracted from green bananas served as the encapsulation medium (Figure 1A). The morphological characteristics and surface properties of RS particles and RS-encapsulated probiotics were examined (Figure 1B). Native RS granules exhibited a smooth surface with an irregular oblate shape and overall integrity (lower-right panel of Figure 1B(d)). Conversely, after encapsulation, the RS microcapsules did not exhibit a distinct shape. Instead, cluster matrices with dents and cavities were observed on the surfaces of the microcapsules (Figure 1B(b–d)). The black arrow in image (b) indicates the presence of probiotic cells encapsulated within this matrix.

**Figure 2:**
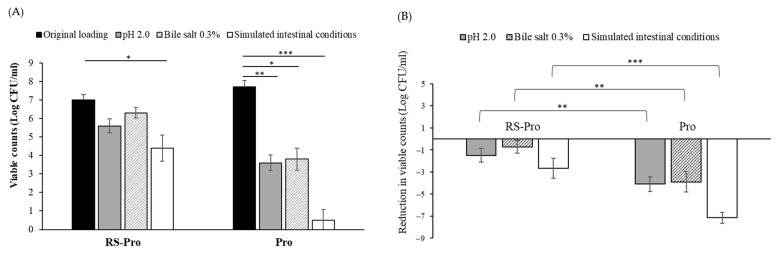
RS-Pro presented tolerance to acid and bile salt. (**A**) The viable counts of probiotics in the probiotic cocktails (Pro) and RS-Pro before and after exposure to acid (gray fill), 0.3% bile salts (cross diagonal), and simulated intestinal conditions (blank) over a 3 h period (or a total of 6 h for simulated intestinal conditions) are presented as a bar chart. Viable counts are expressed in log CFU/mL. Statistical significance is indicated by *, *p* < 0.05, **, *p* < 0.01 and ***, *p* < 0.001 compared with original loading amount, *n* = 3. (**B**) The log reduction in viable cell counts following different treatments compared to the initial inoculation. Data are presented as mean ± SD from 3 independent experiments. Data presented as mean ± SD of 3 independent experiments. **, *p* < 0.01 and ***, *p* < 0.001.

To evaluate the protective role of RS encapsulation in enabling probiotics to reach the colon viably after passing through the gastrointestinal (GI) tract, an in vitro acid and bile tolerance test was conducted. Equal amounts of probiotic cocktails with RS encapsulation (RS-Pro) were exposed to acid, bile salts, and simulated intestinal conditions. Simultaneously, probiotic cocktails without RS encapsulation (Pro) were treated under the same conditions in parallel. The viable counts of probiotics were measured and expressed as log10 CFUs per milliliter (Figure 2A). After 3 h of treatment at pH 2.0, the viable count of bacteria in the RS-Pro group decreased from 7.05 ± 0.29 CFU/mL to 5.56 ± 0.38 CFU/mL, resulting in a 1.49-log reduction. In contrast, the Pro group exhibited a decrease from 7.69 ± 0.36 CFU/mL to 3.59 ± 0.43 CFU/mL, corresponding to a 4.10-log reduction. When exposed to 0.3% bile salts, RS-Pro showed a reduction from 7.05 ± 0.29 CFU/mL to 6.34 ± 0.28 CFU/mL, equivalent to a 0.7-log reduction while Pro decreased from 7.69 ± 0.36 CFU/mL to 3.78 ± 0.59 CFU/mL, equivalent to a 3.9-log reduction (Figure 2B).

Under simulated intestinal conditions over a total 6 h period, the RS-Pro group exhibited a reduction of 2.7 log CFU/mL, whereas the Pro group experienced a reduction of 7.1 log CFU/mL, decreasing from 7.69 ± 0.36 CFU/mL to 0.55 ± 0.58 CFU/mL (Figure 2B). This substantial decline indicates that most free-cell probiotics did not survive. These findings suggest that the encapsulated probiotics (RS-Pro) exhibited significantly enhanced resistance to the adverse effects of bile salts and acidic conditions. Consequently, we assessed the effects of these RS-encapsulated probiotics on mice administered 5-FU.

### 3.2. Probiotics Alongside 5-FU Treatment Altered Composition and Diversity of Gut Microbiota

**Figure 3:**
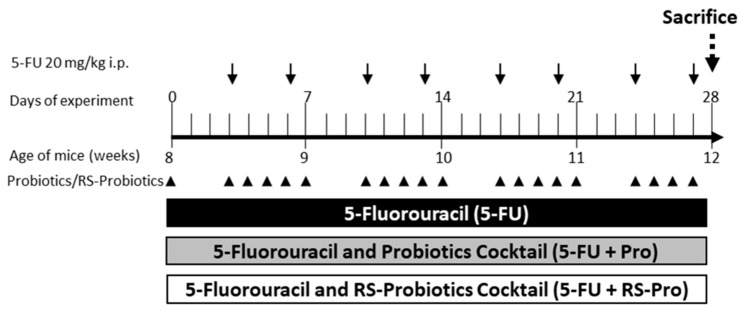
Schematic diagram of animal study. BALB/cByJNarl mice were categorized into three experimental groups. Each group received a 20 mg/kg injection of 5-FU and either probiotics cocktail (Pro or RS-Pro) or no probiotics (5-FU). After 28 days, the animals were euthanized for further analysis.

To evaluate the protective effects of Pro encapsulated with or without RS on mice experiencing 5-FU-induced side effects, 18 mice were administered 5-FU, 5-FU+Pro, and 5-FU+RS-Pro (Figure 3).

**Figure 4:**
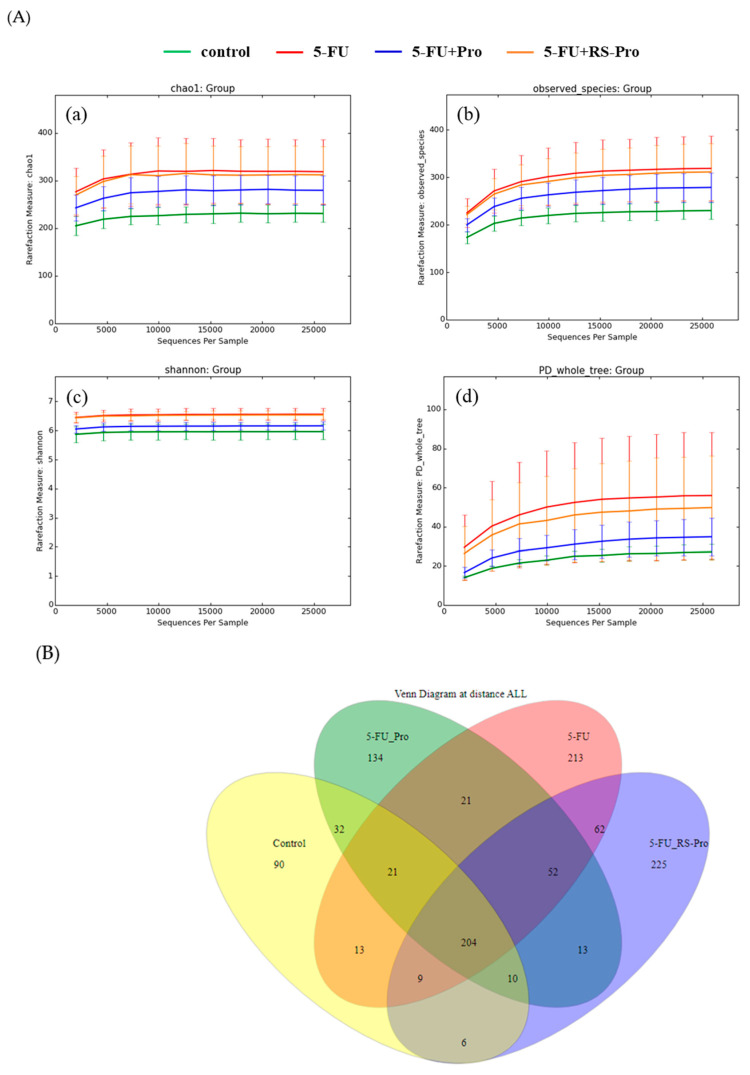
Rarefaction curves and Venn diagram of amplicon sequence variants (ASVs). (**A**) Rarefaction curves representing alpha diversity analysis, including Chao1 index (**a**), observed species (**b**), Shannon index (**c**), and PD whole tree (**d**), illustrating the observed number of species at various sequencing depths. The *y*-axis indicates the average number of ASVs per sample in each group. (**B**) Venn diagram depicting ASVs in different study groups. Yellow, red, green, and blue circles represent different experimental groups, while the intersections reveal shared ASVs among one or more groups. The single-layer zone indicates the number of ASVs specific to each group, with numbers indicating the corresponding species.

To investigate the effects of a probiotic cocktail on changes in gut microbiota following 5-FU treatment, fecal samples were collected from each mouse on day 0 (baseline) and day 27 (after the experiment). The diversity and composition of gut microbiota in the collected fecal samples were analyzed using MiSeq sequencing. The total usable sequences were classified into 13 phyla, 19 classes, 37 orders, 47 families, and 88 genera. Alpha diversity analysis was performed to compare the microbial biodiversity between the groups in terms of their results for the Chao1 index, observed species, Shannon index, and phylogenetic diversity whole tree. The comparison of the control samples (baseline, before treatment) with the treatment samples revealed greater diversity in the treatment samples, as evidenced by the rarefaction curves generated for the four matrices (Figure 4A,B).

A taxonomy summary of the phyla of the mice’s fecal microbiome, presented in Table 1, reveals that 5-FU administration altered the gut microbial profile of the mice. At the phylum level, the relative abundance of Firmicutes in the fecal microbiota of the 5-FU group (72.50 ± 10.50) and 5-FU+RS-Pro group (72.90 ± 11.27) was higher than that of the control group at baseline (46.36 ± 13.50). Furthermore, the proportion of Bacteroidota and Proteobacteria decreased in the 5-FU, 5-FU+Pro, and 5-FU+RS-Pro groups. Prior to the experiment (baseline), the Firmicutes/Bacteroidetes ratio was 0.96 ± 0.45. After the experiment, this ratio was higher in all three groups (5-FU, 4.54 ± 3.27; 5-FU+Pro, 8.45 ± 12.37; and 5-FU+RS-Pro, 4.13 ± 2.76), suggesting that Firmicutes replaced Bacteroidota as the most abundant phylum in fecal microbiota in these groups.

**Figure 5:**
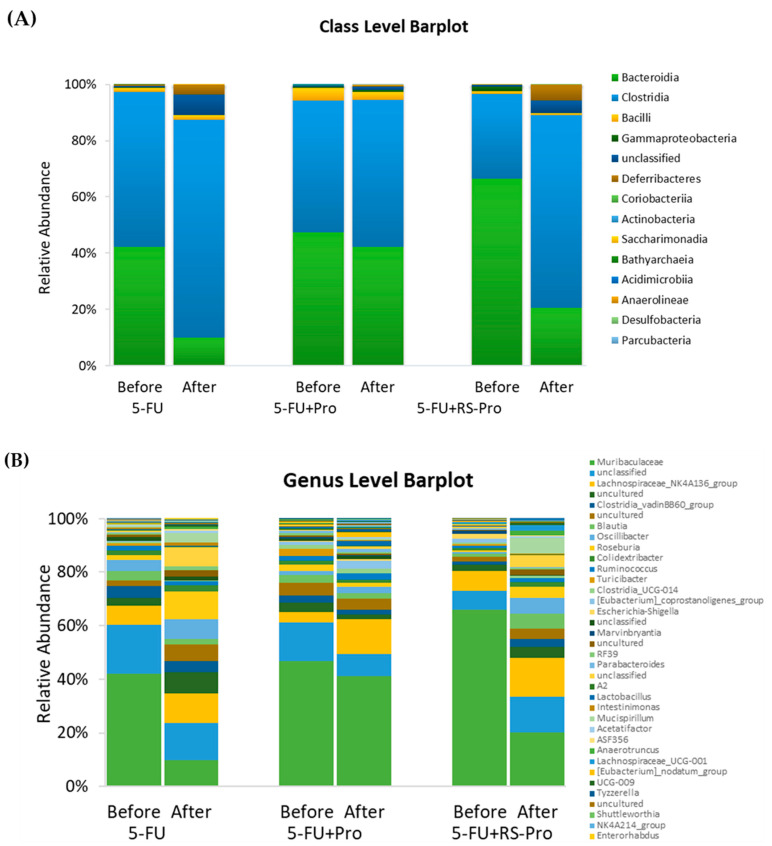
Taxonomic profiles of the fecal bacteria. The figure provides a comparison of the component proportions of the microbiome at the class (**A**) and genus (**B**) levels among the different experimental groups, displaying the taxonomic profiles of fecal bacteria in three groups and illustrating the effects of RS probiotics on 5-FU-induced changes in the relative abundances of gut microbiota. Fecal samples were collected both before (day 0) and after (day 27) the experiment.

In a comparison of the classes of fecal microbiota before and after treatment in the experimental groups, significant decreases were identified with respect to the relative abundance of Bacteroidia, which decreased from 42.21% to 9.9% in the 5-FU group and from 66.5% to 20.5% in the 5-FU+RS-Pro group. By contrast, the relative abundance of Clostridia increased from 55.0% to 77.7% in the 5-FU group and from 30.2% to 68.2% in the 5-FU+RS-Pro group (Figure 5A). Additionally, after 28 days of treatment, the relative abundance of Escherichia–Shigella decreased from 1.7% to 0.023% in the 5-FU+RS-Pro group and slightly increased from 0.4% to 0.6% in the 5-FU+Pro group. In the 5-FU group, the relative abundance of Escherichia–Shigella was only 0.03% before 5-FU administration, and it was not observed after the experiment; this finding indicates that it was not a dominant bacterium (Figure 5B).

Alpha diversity analysis based on ASVs was conducted using DADA2. The 5-FU-treated mice exhibited increased alpha diversity (measured on the basis of the Chao1 index, observed species, Shannon index, and phylogenetic diversity whole tree) relative to the control (baseline) group (Figure 4A). The total richness across all groups was 1105 (control group, 385 species; 5-FU group, 595 species; 5-FU+Pro group, 487 species; 5-FU+RS-Pro group, 581 species; Figure 4B).

**Figure 6:**
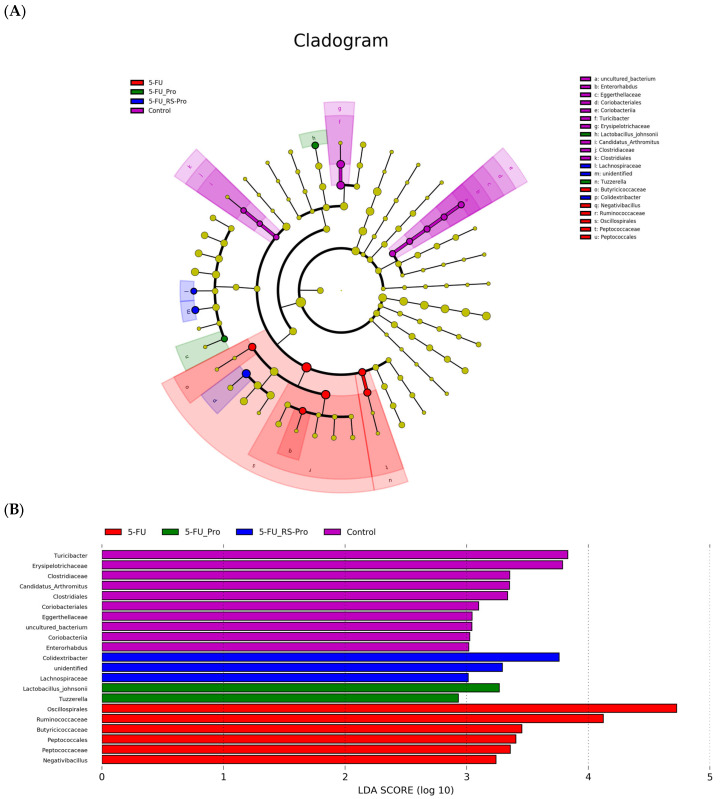
Cladogram and histogram of linear discriminant analysis effect size (LEfSe) among the different groups: control, 5-FU, 5-FU+Pro, and 5-FU+RS-Pro groups. In the cladograms (**A**), taxonomic cladogram representation obtained from LEfSe analysis of 16S sequences highlights statistically significant differences between groups. Different colors of nodes indicate microbial groups that play significant roles: purple nodes in the control group, red nodes in the 5-FU group, green nodes in the 5-FU+Pro group, and blue nodes in the 5-FU+RS-Pro group. The histogram (**B**) displays linear discriminant analysis (LDA) scores assessing the effect size of each differentially abundant bacterial taxa between groups.

A histogram of linear discriminant analysis (LDA) value distribution and an evolutionary branch graph generated through LDA effect size (LEfSe) analysis are shown in Figure 6. LEfSe scores were used to identify biomarkers for distinguishing the groups on the basis of relative abundance; in the results, colors are used to indicate the branch of the phylogenetic tree that most markedly represents a given group. The LEfSe analysis results revealed distinct patterns in the microbial composition between the groups. Specifically, the orders Clostridiales and Coriobacteriales were more abundant in the control group, whereas the orders Peptococcales and Oscillospirales were more prevalent in the 5-FU group (Figure 6A). The obtained LDA scores underscored the substantial differences in gut microbiota biomarkers between the groups. Notably, the main genera were *Candidatus* Arthromitus, *Turicibacter*, and *Enterorhabdus* in the control group; *Negativibacillus* in the 5-FU group; *Tuzzerella* in the 5-FU+Pro group; and *Colidextribacter* in the 5-FU+Rs-Pro group (Figure 6B).

### 3.3. Supplementation with Probiotics Demonstrated Potential to Attenuate 5-FU-Induced Intestinal Damage and Hematologic Toxicity

**Figure 7:**
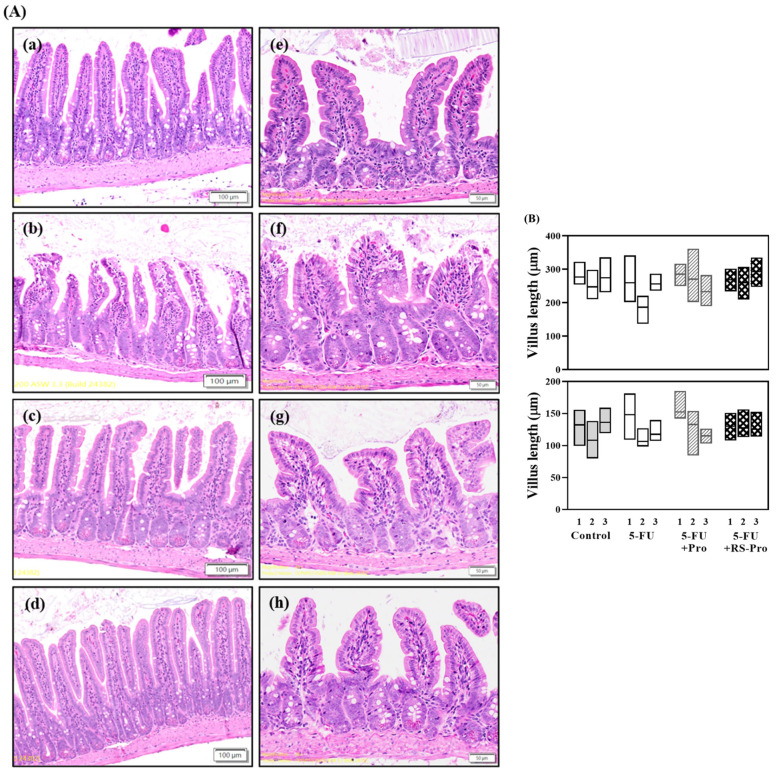
Histological analysis of the small intestine following 5-FU and probiotics cocktail administration. (**A**) Representative histological images of small intestine fragments stained with hematoxylin and eosin from various experimental groups are presented. Additional mice of the same age were employed as normal controls. In panel (**a**–**e**), it is evident that normal control mice exhibited the integrity of the intestinal mucosa, with normal villi and crypts. Mice treated with 5-FU (20 mg/kg twice weekly) (panel (**b**,**f**)) exhibited loss of intestinal mucosal integrity, shortened and blunted villi, and inflammatory cell infiltration in the lamina propria. Mice treated with Pro and RS-Pro (panel (**c**,**d**,**g**,**h**)) showed alleviated 5-FU-induced histological changes, with preserved villi. Scale bars = 100 μm (panel (**a**–**d**)) and 50 μm (panel (**e**–**h**)). (**B**) Measurements of villus height (**upper**) and crypt depth (**lower**) in intestinal segments. The box graph represents the maximum, median, and minimum.

To evaluate the role of Pro and RS-Pro in 5-FU-induced colitis, the histopathological morphology of mouse small intestine segments was observed and the lengths of villi and crypts were quantified as shown in Figure 7A,B. Same-age mice served as normal control. Compared to the normal control, histological analysis revealed that 5-FU treatment damaged intestinal epithelial cells and disrupted the integrity of the intestinal barrier, resulting in the blunting and shortening of villi and the presence of inflammatory in-filtrates (Figure 7A(b,f)). Treatment with Pro (5-FU+Pro group) or RS-Pro (5-FU+RS-Pro group) alleviated these adverse effects, leading to an improvement in the appearance of villi and crypts. Notably, slight abnormalities were still observed in the 5-FU+Pro group relative to the 5-FU+RS-Pro group. This finding suggests that RS-Pro provides increased protection against the damage caused by 5-FU.

The systemic effects of each treatment were evaluated through clinical observations, body weight measurements, major organ (spleen, liver, and kidney) weight measurements, and blood cell count (CBC) assessments. No adverse signs were observed in the mice. No significant changes in body weight and organ weight were observed among the groups, except the spleen weights of mice treated with 5-FU+Pro were less than mice treated with 5-FU (*p* < 0.05, Supplementary Figure S1). The results of CBC showed that the peripheral white blood cells (WBCs), red blood cells (RBCs), hemoglobin (HGB), reticulocytes (RET), neutrophils (NEUT), monocytes (MONO) and eosinophils (EOS) were significantly decreased in mice received 5-FU treatment for 4 weeks. Partial detection items (e.g., RBC, RET) were relatively decreased in mice that received 5-FU+Pro or 5-FU+RS-Pro (Table 2). However, there was no significant difference between 5-FU+Pro vs. 5-FU or 5-FU+RS-Pro vs. 5-FU, respectively, except the RET (Supplementary Table S1).

## 4. Discussion

In recent decades, probiotics and prebiotics have garnered attention for their potential health benefits, including immune system modulation, infection resistance, and anticancer effects [2,14]. However, for probiotics to exert their positive effects, they must survive the harsh conditions of the upper gastrointestinal tract and reach their intended site of action in a viable state. Studies have demonstrated that the viability of probiotics may be compromised in environments with high gastric acidity. Microencapsulation techniques have emerged as an effective means for enhancing probiotic survival under these challenging conditions [20]. Our study used RS as the encapsulation material, and our results indicated that encapsulation with RS significantly increased the viability of probiotics in gastric acid, bile salts (0.3%), and under simulated intestinal conditions.

Notably, we implemented a 5-FU dosage regimen aimed at mimicking the long-term concomitant use of probiotics. Crucially, in clinical settings, 5-FU dosing regimens may vary on the basis of indications. Although our regimen (20 mg/kg of 5-FU twice weekly for 28 days) was selected to simulate prolonged treatment, other studies have used higher 5-FU doses to induce acute and severe intestinal mucositis. However, these high-dose treatments often resulted in severe side effects and even high mortality rates [35].

Research has highlighted the effects of gut microbiota dysbiosis induced by 5-FU treatment [11]. Alterations in gut microbiota composition can affect the function of the mucosal immune system, potentially leading to intestinal inflammation [36]. Our study confirmed that 5-FU administration altered the gut microbiome in mice, reducing their overall alpha diversity and changing their microbial composition. Specifically, the abundance of Firmicutes increased at the phylum level, indicating dysbiosis. Increasing evidence indicated that the probiotics and prebiotics might modulate the microbiome dysbiosis caused by 5-FU treatment; however, the results vary depending on the model, the dosing, and the treatment plan [37,38]. In our current study, 5-FU treatment increased Firmicutes/Bacteroidota ratio (F/B) by approximately 4 to 8 times compared to the control group (Table 1). The phylum level of the F/B ratio was similar between the 5-FU and 5-FU+RS-Pro groups, indicating that RS-Pro supplementation seemed not to restore the disrupted microbiota at the phylum level. However, Figure 6 showed that cladogram analysis revealed a major impact of the 5-FU treatment on lower taxonomic ranks. Our data also indicated that the *Muribaculaceae*, a family of bacteria within the order of *Bacteroidales*, was more abundance in 5-FU+RS-Pro among groups.

The 5-FU treatment resulting in the blunting and shortening of villi and the presence of inflammatory infiltrates might be caused by dysbiosis of gut microbiota and were observed in this study (Figure 7). The results are consistent with previous research [10]. Probiotics and prebiotics are commonly used as therapeutic alternatives to prevent or ameliorate 5-FU-induced gut microbiota dysbiosis [39]. In this study, the Pro and RS-Pro partially restored the dysbiosis of microbiota caused by 5-FU. For instance, the class of Clostridia was relatively more abundant in 5-FU+RS-Pro than in 5-FU+Pro. Trachsei et al. demonstrated that the abundance of Clostridia was increased after the intake of resistant starch. They also indicated that animals fed with resistant starch increased the short-chain fatty acid (SCFA) concentrations in cecum and feces, potentially modulating microbiota and host immune status [40]. Whether RS-Pro, composed of resistant starch and probiotics, has similar or synergistic effects on host immune activity requires further investigation.

Our macroscopic evaluation revealed that the average body weight of the mice in all three groups slightly increased by the end of our experiment, which is consistent with the findings for similar treatments [36]. Some studies have reported significant body weight loss and reduced food consumption in response to high-dosage 5-FU treatment, which induces acute intestinal mucositis [34,35,41]. This discrepancy in results may be attributed to differences in experimental design, dosages, and mouse strains used. Our model may serve as a potential way to evaluate the impact of chronic intestinal damage on long-term probiotic intake.

A common side effect of 5-FU is a reduction in white blood cell count, which increases the risk of infections because of its effect on the hematopoietic system. This trend was observed in our study, as evidenced by the marked reduction in leukocytes, red blood cells, and neutrophils, and an increase in platelets, in the 5-FU-treated mice. Notably, treatment with RS-Pro or Pro led to a recovery in peripheral blood counts and mitigated the hematopoietic effects of 5-FU.

The administration of 5-FU has been reported to induce intestinal injury, particularly intestinal mucositis [42]. In our study, administration of 5-FU caused intestinal damage, including disrupted mucosal integrity, decreased villus length, blunted villi, and increased inflammatory cell infiltration in the lamina propria. This finding is consistent with those of other studies that demonstrated the damaging effects of 5-FU on the intestinal mucosa. Importantly, RS-Pro treatment alleviated the severity of 5-FU-induced intestinal damage. Although the Pro group also improved 5-FU-induced intestinal damage, slight abnormalities persisted in the intestinal morphology of the mice. These results suggest that RS-Pro may enhance the delivery and efficacy of probiotics by providing protection through resistant starch encapsulation.

In addition to gut microbiota dysbiosis and intestinal injury, leucopenia is one hallmark of 5-FU-induced adverse effects [43]. Similarly, our study observed a marked reduction in leukocytes, RBCs, HGB, and RET in the 5-FU-treated mice. Despite the fact that there is no statistically significant difference between the 5-FU+Pro vs. 5-FU or 5-FU+RS-Pro vs. 5-FU groups, RET and RBCs were significantly decreased in mice that received 5-FU+Pro and 5-FU+RS-Pro treatments, respectively.

In summary, the present preliminary study supports the idea that resistant starch encapsulation enhances the acid and bile salt tolerance of probiotic cocktails during gastrointestinal transit. Supplementation with RS-encapsulated probiotics demonstrates potential in mitigating long-term 5-FU-induced abnormalities, such as gut microbiota dysbiosis, intestinal damage, and leucopenia. These findings underscore the potential utility of RS-encapsulated probiotics as a supplement to standard chemotherapy, potentially enhancing therapeutic outcomes and minimizing treatment-related side effects. Further research is needed to explore the clinical applications and underlying mechanisms of these effects.

## Figures and Tables

**Table 1:** The relative abundance of the top 10 gut microbiota distributed at the phylum level after 5-FU and probiotics cocktail administration.

Phyla Relative Abundance (%)	Control	5-FU	5-FU+Pro	5-FU+RS-Pro
Bacteroidota	52.01 ± 12.77	22.57 ± 14.49	29.72 ± 22.27	22.53 ± 11.78
Firmicutes	46.36 ± 13.50	72.50 ± 10.50	67.51 ± 20.34	72.90 ± 11.27
Proteobacteria	0.72 ± 0.86	0.03 ± 0.04	0.24 ± 0.34	0.02 ± 0.02
unclassified	0.49 ± 0.09	2.96 ± 3.70	0.83 ± 0.41	1.99 ± 2.09
Deferribacterota	0.25 ± 0.26	1.71 ± 1.76	1.59 ± 2.0	2.49 ± 2.90
Actinobacteriota	0.14 ± 0.09	0.09 ± 0.16	0.02 ± 0.02	0.02 ± 0.02
Patescibacteria	0.03 ± 0.02	0.13 ± 0.03	0.09 ± 0.13	0.04 ± 0.03
Crenarchaeota	0.00 ± 0.00	0.00 ± 0.00	0.00 ± 0.00	0.01 ± 0.01
Acidobacteriota	0.00 ± 0.00	0.00 ± 0.00	0.00 ± 0.00	0.00 ± 0.00
Chloroflexi	0.00 ± 0.00	0.00 ± 0.00	0.00 ± 0.01	0.00 ± 0.00
Firmicutes/Bacteroidota (F/B)	0.96 ± 0.45	4.54 ± 3.27	8.45 ± 12.37	4.13 ± 2.76

**Table 2:** Peripheral blood cell counts (CBC) in mice before and after receiving different treatments.

Items	Unit	5-FU			5-FU+Pro			5-FU+RS-Pro		
		Before (Day 0)	After (Day 26)	*p*	Before (Day 0)	After (Day 26)	*p*	Before (Day 0)	After (Day 26)	*p*
WBCs	×10^3^ cells/µL	11.13 ± 2.28	8.77 ± 0.73	0.04	11.82 ± 2.28	9.55 ± 1.95	0.20	12.06 ± 2.15	10.65 ± 2.16	0.15
RBCs	×10^6^ cells/µL	10.80 ± 0.23	10.23 ± 0.17	<0.001	11.01 ± 0.35	10.52 ± 0.31	0.09	10.80 ± 0.26	10.20 ± 0.25	0.02
HGB	g/dL	16.75 ± 0.33	16.13 ± 0.30	<0.01	17.07 ± 0.54	16.55 ± 0.41	0.20	16.80 ± 0.41	16.08 ± 0.39	0.06
PLT	×10^3^ cells/µL	723.17 ± 57.59	862.67 ± 46.82	<0.001	727.00 ± 122	854.50 ± 57.13	0.08	702.17 ± 27.95	825.00 ± 97.98	0.01
abs_ret	×10^3^ cells/µL	540.88 ± 90.53	416.77 ± 41.77	<0.01	524.80 ± 42.36	451.63 ± 40.4	0.04	493.93 ± 47.6	447.53 ± 48.02	0.06
abs_neuts	×10^3^ cells/µL	2.35 ± 0.75	1.54 ± 0.36	0.03	2.07 ± 0.62	1.75 ± 0.55	0.37	1.86 ± 0.42	2.02 ± 0.48	0.60
abs_lymphs	×10^3^ cells/µL	8.35 ± 2.28	6.94 ± 0.55	0.17	9.29 ± 1.84	7.49 ± 1.48	0.20	9.73 ± 1.7	8.27 ± 1.75	0.05
abs_monos	×10^3^ cells/µL	0.25 ± 0.05	0.14 ± 0.02	0.01	0.22 ± 0.04	0.16 ± 0.07	0.09	0.27 ± 0.1	0.21 ± 0.04	0.12
abs_eos	×10^3^ cells/µL	0.17 ± 0.05	0.14 ± 0.04	0.01	0.23 ± 0.11	0.15 ± 0.04	0.16	0.19 ± 0.04	0.15 ± 0.08	0.26
abs_basos	×10^3^ cells/µL	0.01 ± 0.01	0.01 ± 0.01	0.79	0.00 ± 0.01	0.01 ± 0.01	0.36	0.01 ± 0.01	0.01 ± 0.01	0.20

WBCs, white blood cells; RBCs, red blood cells; HGB, hemoglobin; PLT, platelet; abs, absolute; ret, reticulocytes; neuts, neutrophil; monos, monocytes; lymphs, lymphocytes; eos, eosinophils; basos, basophils; *p*, significant difference between before vs. after from paired *t*-test, *n* = 6/group. Data are presented as mean ± standard deviation (SD).

## Data Availability

The original contributions presented in the study are included in the article/Supplementary Materials, further inquiries can be directed to the corresponding authors.

## Supplementary Materials

The following supporting information can be downloaded at: https://www.mdpi.com/article/10.3390/biomedicines12081912/s1, Figure S1: Trends in average body weight change and organ weight in different experimental groups; Table S1: The p value to compare peripheral blood cell counts of 5-FU+Pro versus 5-FU or 5-FU+RS-Pro versus 5-FU using 2-way ANOVA followed by Dunnett’s multiple comparisons test.
