# Supplementary material for: Effects of Resistant-Starch-Encapsulated Probiotic Cocktail on Intestines Damaged by 5-Fluorouracil
Source: Biomedicines. 2024 Aug 20;12(8):1912. doi: 10.3390/biomedicines12081912 (PMC11351836; doi:10.3390/biomedicines12081912)
Supplement: Supplementary file 1 [file biomedicines-12-01912-s001.zip › biomedicines-3091791-supplementary.pdf]

(A)

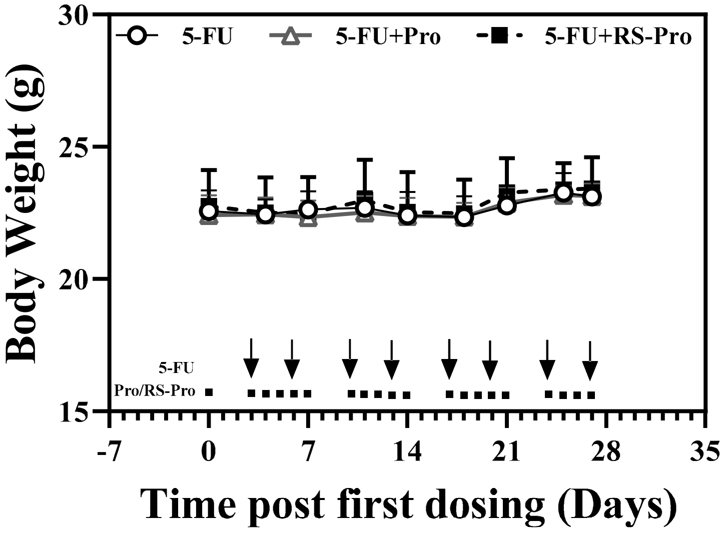

(B)

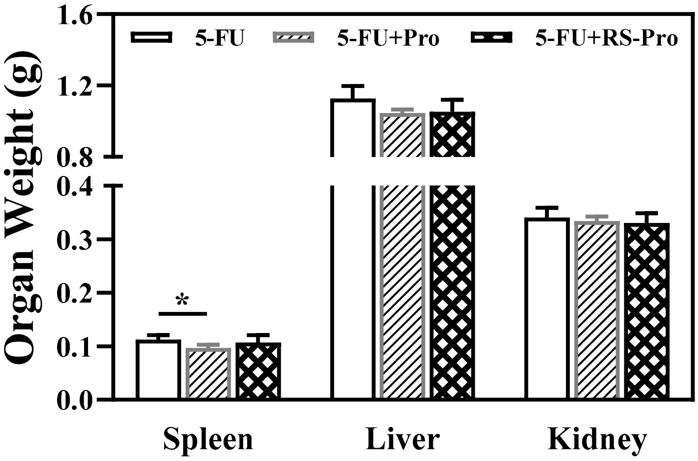

Supplementary Figure S1. Trends in average body weight change and organ weight in different experimental groups

(A) Body weights were monitored throughout the treatment period from day 0 to day 27. (B) Organ weights of the spleen, liver, and kidney in the different experimental groups. Statistical analyses were conducted using one-way ANOVA with Tukey's multiple comparisons test. * $p < 0.05$.

Supplementary Table S1. The *p* value to compare peripheral blood cell counts of 5-FU+Pro versus 5-FU or 5-FU+RS-Pro versus 5-FU using 2-way ANOVA followed by Dunnett's multiple comparisons test.

Items	5-FU+Pro v.s. 5-FU	5-FU+RS-Pro v.s. 5-FU
WBC	> 0.999	0.997
RBC	> 0.999	> 0.999
HGB	> 0.999	> 0.999
PLT	0.612	0.402
abs_ret	0.001	< 0.0001
abs_neuts	0.999	0.997
abs_lymphs	0.999	> 0.999
abs_monos	> 0.999	> 0.999
abs_eos	> 0.999	> 0.999
abs_basos	> 0.999	> 0.999

WBC, white blood cells; **RBC,** red blood cells; **HGB,** hemoglobin; **PLT,** platelet; **abs,** absolute; **ret,** reticulocytes; **neuts,** neutrophil; **monos,** monocytes; **lymphs,** lymphocytes; **eos,** eosinophils; **basos,** basophils; **v.s.,** versus
